# Comparison between Phase-Shift Full-Bridge Converters with Noncoupled and Coupled Current-Doubler Rectifier

**DOI:** 10.1155/2013/621896

**Published:** 2013-12-05

**Authors:** Cheng-Tao Tsai, Jye-Chau Su, Sheng-Yu Tseng

**Affiliations:** ^1^Department of Electrical Engineering, National Chin-Yi University of Technology, Taichung 41170, Taiwan; ^2^Department of Electronic Engineering, National Chin-Yi University of Technology, Taichung 41170, Taiwan; ^3^Department of Electrical Engineering, Chang-Gung University, Tao-Yuan 33344, Taiwan

## Abstract

This paper presents comparison between phase-shift full-bridge converters with noncoupled and coupled current-doubler rectifier. In high current capability and high step-down voltage conversion, a phase-shift full-bridge converter with a conventional current-doubler rectifier has the common limitations of extremely low duty ratio and high component stresses. To overcome these limitations, a phase-shift full-bridge converter with a noncoupled current-doubler rectifier (NCDR) or a coupled current-doubler rectifier (CCDR) is, respectively, proposed and implemented. In this study, performance analysis and efficiency obtained from a 500 W phase-shift full-bridge converter with two improved current-doubler rectifiers are presented and compared. From their prototypes, experimental results have verified that the phase-shift full-bridge converter with NCDR has optimal duty ratio, lower component stresses, and output current ripple. In component count and efficiency comparison, CCDR has fewer components and higher efficiency at full load condition. For small size and high efficiency requirements, CCDR is relatively suitable for high step-down voltage and high efficiency applications.

## 1. Introduction

**Figure 1:**
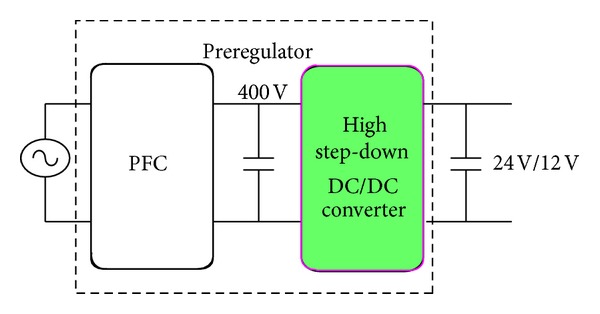
Two-stage structure of preregulator.

In a decentralized power system, the front end ac/dc converter is generally composed of two stages, in which one is a power factor correction (PFC) and the other is an intermediate dc/dc converter, as shown in Figure 1. Most of PFC circuits adopt a boost converter [1], and an intermediate converter is usually with an isolated version [2]. Using a boost converter can achieve a unity power factory, and using an isolated converter can provide galvanic isolation and high output current. In off-line applications, universal ac voltage is always into dc 400 V as a dc bus by boost converters, and an intermediate dc/dc converter converts it to a low voltage bus of 24 V_dc_ or 12 V_dc_. Therefore, for high output current and low output voltage applications, an isolated dc/dc converter is usually required.

**Figure 2:**
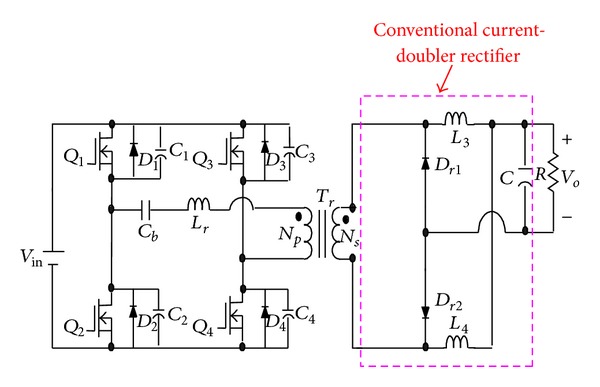
The phase-shift full-bridge converter with conventional current-doubler rectifier.

To achieve low output voltage, high output current, and high efficiency, a phase-shift full-bridge converter with conventional current-doubler rectifier is widely used in medium-high power condition, as shown in Figure 2 [3]. Nevertheless, it still has several limitations. For example, for high step-down voltage conversion, it requires a transformer with high turns ratio or it has to reduce the duty ratio of the switches. A high turns ratio will result in high duty loss and low conversion efficiency, while a low duty ratio will increase input peak current and component stress [4]. The other limitation is that its large external resonant inductor will induce a large circulation current, which will flow through the primary winding of the transformer and the switches during a freewheeling interval. As a result, conduction loss in the switches and copper loss in the transformer are significant. To release the above-mentioned limitations of the conventional full-bridge converter with current-doubler, many approaches have been conducted [3–7]. However, their high step-down voltage ratio still result in extremely low duty ratio, which will induce high peak current through the secondary winding of the isolation transformer and output filter inductors, increasing copper loss and component stresses [8–12].

**Figure 3:**
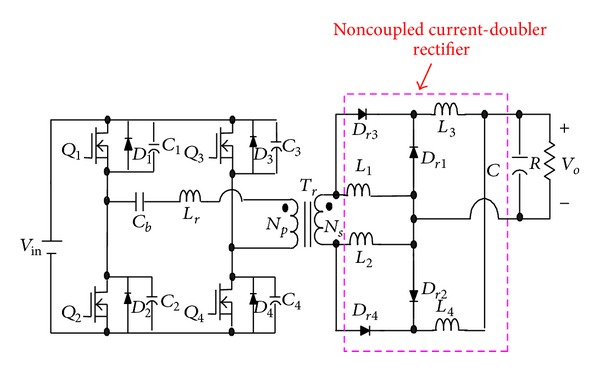
The proposed phase-shift full-bridge converter with NCDR.

**Figure 4:**
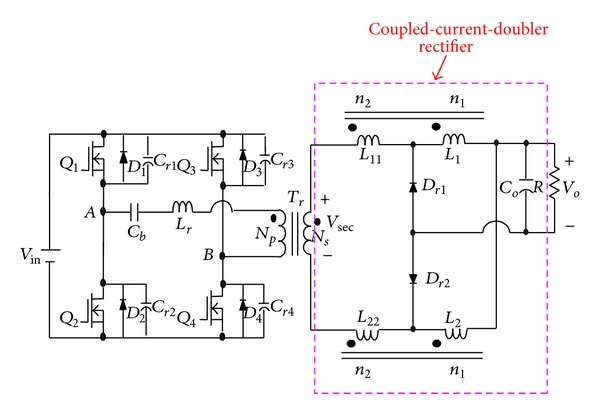
The proposed phase-shift full-bridge converter with CCDR.

To solve the above-mentioned problem, the phase-shift full-bridge converter with NCDR or CCDR is proposed, as shown in Figures 3 and 4 [13]. They can alleviate the drawbacks of extremely low duty ratio and high component stresses. The two proposed improved rectifiers can extend duty ratio of the active switches to reduce the peak current through the secondary winding of the transformer and lower output current ripple. The conversion efficiency can be increased significantly.  Section 2 describes derivation and operational principle of the two proposed improved current-doubler rectifiers.  Section 3 compares the benefits of the two improved current-doubler rectifiers. Power loss and efficiency estimation are described in Section 4. Experimental results obtained from a 500 W phase-shift full-bridge converter with NCDR and CCDR are presented in Section 5. Finally, a conclusion is given in Section 6.

## 2. Derivation of Improved Current-Doubler Rectifiers

With duality method, NCDR can be derived from a voltage-quadrupler circuit, and coupled-doubler can be derived from a voltage-doubler circuits. In the following, derivations of both improved current-doubler rectifiers are described in details.

### 2.1. Derivation of NCDR

**Figure 5:**
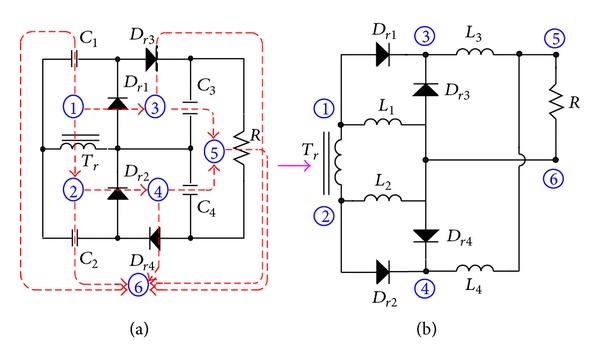
Derivation of NCDR from a voltage-quadrupler based on duality principle: (a) voltage-quadrupler and (b) NCDR.

Derivation of NCDR is based on a conventional voltage-quadrupler circuit, as shown in Figure 5(a). According to duality principle, meshes of the voltage quadrupler are replaced with nodes, and capacitors are replaced with inductors, while diodes are with no change, yielding the proposed NCDR as shown in Figure 5(b).

### 2.2. Derivation of CCDR

**Figure 6:**
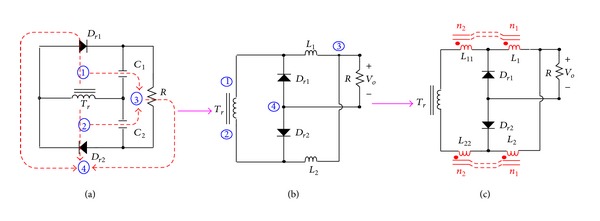
Derivation of CCDR from a voltage-doubler based on duality principle: (a) voltage-doubler, (b) conventional current-doubler rectifier, and (c) CCDR.

Similarly, derivation of CCDR is based on a conventional voltage-doubler circuit, as shown in Figure 6(a). According to duality principle, meshes of the voltage doubler are replaced with nodes, and capacitors are replaced with inductors, while diodes are with no change, yielding the conventional current-doubler rectifier as shown in Figure 6(b). Utilizing coupled inductor concept, the output filter inductors can be extended to the coupled ones, as shown in Figure 6(c).

## 3. Operational Principles of NCDR and CCDR

For NCDR and CCDR, each of which has its own merits and demerits. To have an objective judgment, operational principles of NCDR and CCDR are briefly described as follows.

### 3.1. Operational Principle of NCDR

**Figure 7:**
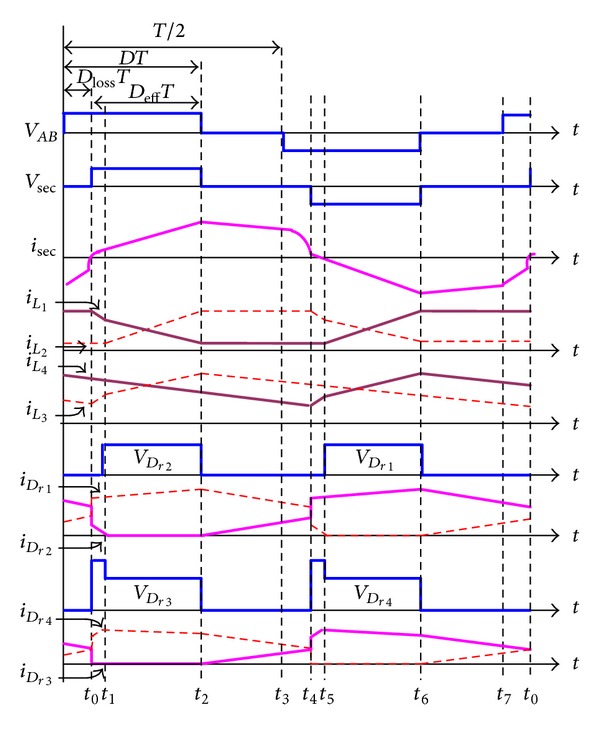
Key waveforms of the proposed phase-shift full-bridge converter with NCDR.

**Figure 8:**
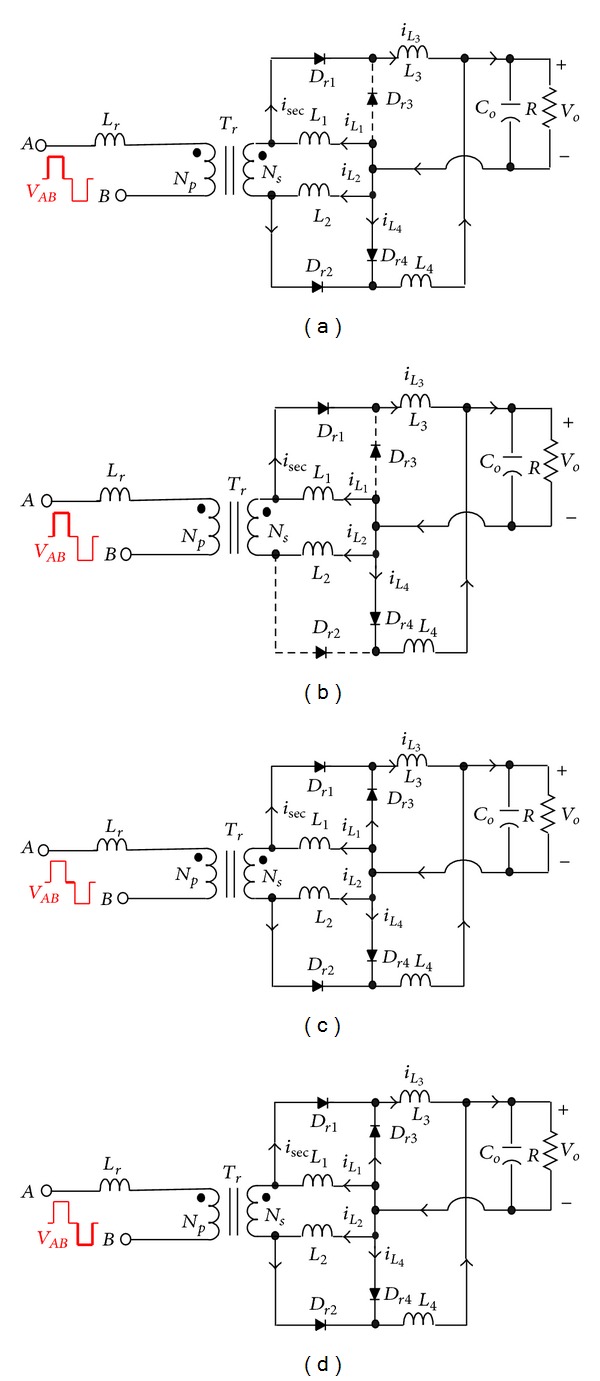
Operational modes of the proposed full-bridge phase-shift converter with NCDR: (a) mode 1, (b) mode 2, (c) mode 3, and (d) mode 4.

In Figure 3, the proposed phase-shift full-bridge converter with NCDR under continuous inductor current operation can be divided into four major operating modes over a half switching cycle.  Figure 7 shows conceptual voltage and current waveforms relative to key components of NCDR. *D*_eff_ and *D*_loss_ are denoted as the effective and loss duty ratios, respectively. *V*_*AB*_ is the voltage across the resonant inductor *L*_*r*_ and the isolation-transformer primary winding, *V*_sec_ is the voltage across the isolation-transformer secondary winding, *i*_sec_ is the secondary current, *i*_*L*_1__ and *i*_*L*_2__ are the current of the energy inductors, *i*_*L*_3__ and *i*_*L*_4__ are the current of the output filter inductors, and *i*_*D*_1__ ~ *i*_*D*_4__ are the current of the rectifier diodes. To simplify description of the steady-state operational modes, the phase-shift full-bridge converter will not be discussed in this section. Only the proposed NCDR is analyzed. Under continuous inductor current operation, four major operating modes of the NCDR are identified over a half switching cycle.  Figure 8 shows equivalent circuits of the NCDR operational modes.

*Mode 1 (Figure 8(a),* *t*_0_ ≤ *t* < *t*_1_). At time *t*_0_, a positive voltage *V*_sec_ crosses the secondary winding of transformer *T*_*r*_. First of all, diode *D*_*r*3_ is reversely biased and *D*_*r*1_, *D*_*r*2_, and *D*_*r*4_ are conducting. During this interval, inductor current *i*_*L*_3__ flowing through the path *V*_*o*_-*L*_2_-*V*_sec_-*D*_*r*1_-*L*_3_ is linearly increased, and inductor currents *i*_*L*_1__ and *i*_*L*_4__are linearly decreased. 

*Mode 2 (Figure 8(b),* *t*_1_ ≤ *t* < *t*_2_). At time *t*_1_, the secondary current *i*_sec_ is equal to inductor current *i*_*L*_3__, and diode *D*_*r*2_ is reversely biased. Inductor current *i*_*L*_2__ = *i*_*L*_3__ flowing through the path *V*_sec_-*D*_*r*1_-*L*_3_-*V*_*o*_-*L*_2_ is linearly increased, while the energy stored in inductor *L*_1_ and *L*_4_ will be released through the rectifier diode *D*_*r*1_ and *D*_*r*4_ to the load, respectively.

*Mode 3 (Figure 8(c),* *t*_2_ ≤ *t* < *t*_3_). When voltage *V*_sec_ drops to zero at time *t*_2_, all of the diodes (*D*_*r*1_ ~ *D*_*r*4_) are conducting. During this interval, the inductor current *i*_*L*_3__ flowing through two paths *V*_*o*_-*D*_*r*3_-*L*_3_ and *V*_*o*_-*L*_2_-*V*_sec_-*D*_*r*1_-*L*_3_ and the inductor current *i*_*L*_4__ flowing through *V*_*o*_-*D*_*r*4_-*L*_4_ are linearly decreased.

*Mode 4 (Figure 8(d),* *t*_3_ ≤ *t* < *t*_4_). At time *t*_3_, a negative voltage *V*_*AB*_ will cross the resonant inductor *L*_*r*_ and the primary winding of transformer *T*_*r*_, since rectifier diode currents *i*_*D*_*r*3__ and *i*_*D*_*r*4__ have not been commutated completely yet. Therefore, all of the diodes (*D*_*r*1_ ~ *D*_*r*4_) are maintained conducting, while inductor currents *i*_*L*_3__ and *i*_*L*_4__ are maintained discharging to the load.

At time *t*_4_, rectifier diode currents *i*_*D*_*r*3__ and *i*_*D*_*r*4__ have been commutated completely. Then, a positive voltage *V*_sec_ crosses the secondary winding of transformer *T*_*r*_. This ends a half switching cycle operation.

### 3.2. Operational Principle of CCDR

**Figure 9:**
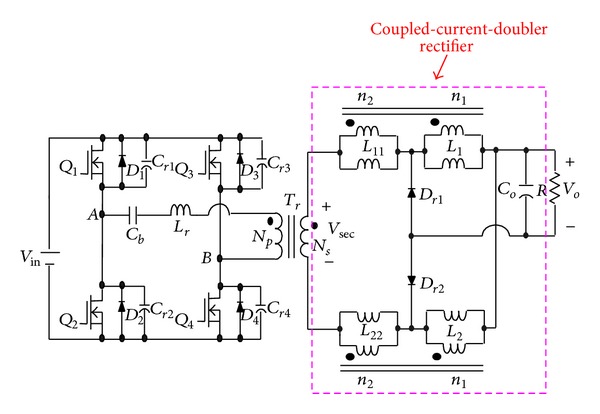
Each coupled inductor individually functions as a transformer for CCDR.

**Figure 10:**
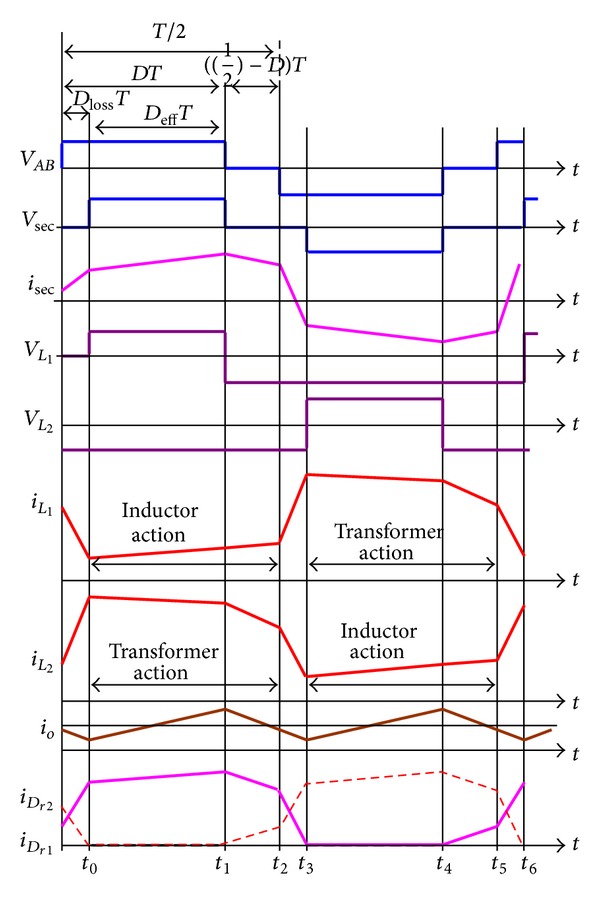
Key waveforms of phase-shift full-bridge converter with CCDR.

In Figure 4, each coupled inductor individually functions as a tapped inductor or a transformer during one switching cycle. In other words, the upper coupled-inductor is charged during the charging period, which functions as a tapped inductor, while the lower coupled-inductor functions as a transformer. Therefore, Figure 4 can be redrawn as shown in Figure 9. The proposed phase-shift full-bridge converter with CCDR under continuous inductor current operation can be divided into three major operating modes over a half switching cycle.  Figure 10 shows conceptual voltage and current waveforms relative to key components of the converter. *D*_eff_ and *D*_loss_ are denoted as the effective and lost duty ratios, respectively. *V*_*AB*_ is the voltage across the resonant inductor and the isolation-transformer primary winding, *V*_sec_ is the voltage across the isolation-transformer secondary winding, *i*_sec_ is the secondary current, *i*_*L*_ and *V*_*L*_ are the current and voltage of the coupled-inductor winding *n*_1_, *i*_*D*_*r*__ and *V*_*D*_*r*__ are the current and voltage of the rectifier diode, and *i*_*o*_ is the output current. The circuit operation is explained as follows.

**Figure 11:**
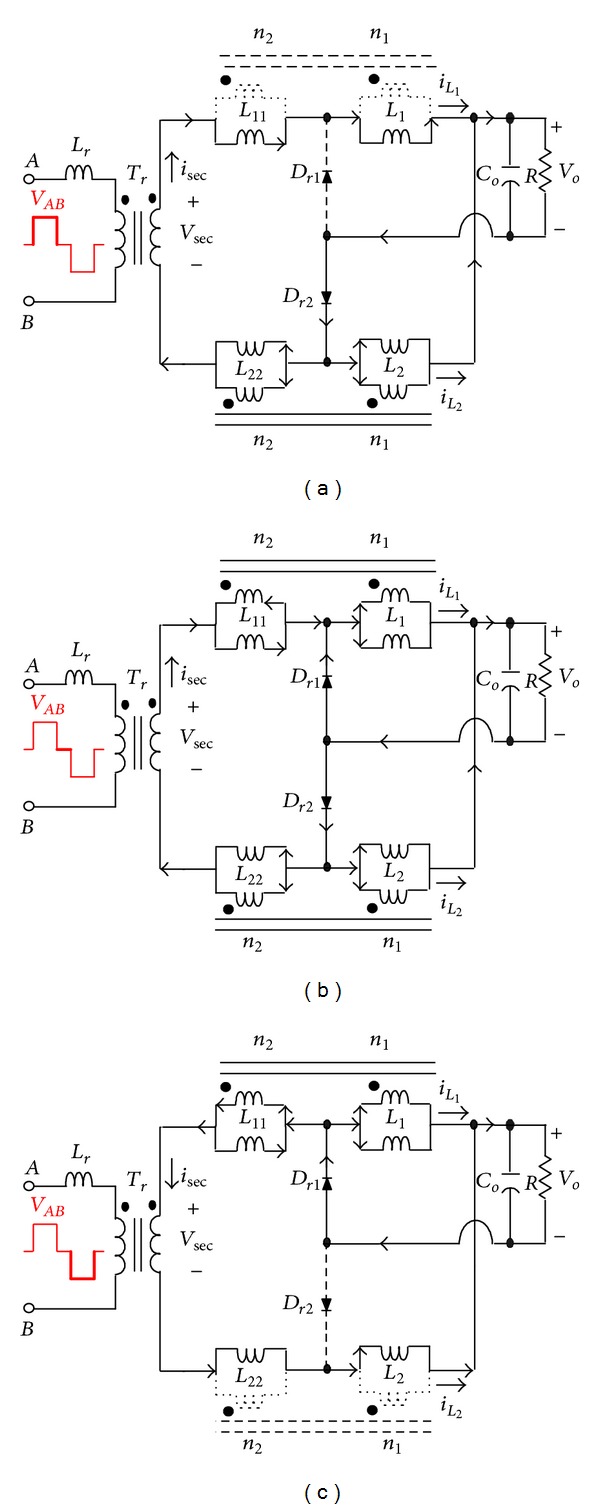
Equivalent circuit modes of the CCDR operating over a half switching cycle.

*Mode 1 (Figure 11(a),* *t*_0_ ≤ *t* < *t*_1_). At time *t*_0_, currents *i*_*D*_*r*1__ and *i*_*D*_*r*2__ are commutated completely. Then, a positive voltage *V*_sec_ crosses the secondary winding of transformer *T*_*r*_; diode *D*_*r*1_ is reversely biased, and inductor current *i*_*L*_1__ flowing through the path of *V*_*o*_-*D*_*r*2_-*L*_22_-*V*_sec_-*L*_11_-*L*_1_ increases linearly. During this interval, the energy stored in inductor *L*_22_ will be released to the load through coupled inductor *L*_2_, and inductor current *i*_*L*_2__ flowing through the path of *V*_*o*_-*D*_*r*2_ is decreased. Meanwhile, inductors *L*_11_ and *L*_1_ function as a tapped inductor, while inductors *L*_22_ and *L*_2_ are coupled to function as a transformer *T*.

*Mode 2 (Figure 11(b),* *t*_1_ ≤ *t* < *t*_2_). When voltage *V*_sec_ drops to zero at time *t*_1_, the energy stored in inductor *L*_22_ is no longer released to the load through coupled inductor *L*_2_. Therefore, inductor current *i*_*L*_2__ will be gradually decreased, rectifier diodes *D*_*r*1_ and *D*_*r*2_ are conducted, and rectifier diode currents *i*_*D*_*r*1__ and *i*_*D*_*r*2__ begin commutating. During this free-wheeling interval, inductor currents *i*_*L*_1__ and *i*_*L*_2__ decrease linearly.

*Mode 3 (Figure 11(c),* *t*_2_ ≤ *t* < *t*_3_ *).* At time *t*_2_, a negative voltage *V*_*AB*_ will cross resonant inductor *L*_*r*_ and the primary winding of transformer *T*_*r*_, since rectifier diode currents *i*_*D*_*r*1__ and *i*_*D*_*r*2__ have not been commutated completely yet. Therefore, the two rectifier diodes *D*_*r*1_ and *D*_*r*2_ are maintained conducting, while inductors *L*_1_ and *L*_2_ are discharged through diodes *D*_*r*1_ and *D*_*r*2_, respectively.

When currents *i*_*D*_*r*1__ and *i*_*D*_*r*2__ are commutated completely at time *t*_3_, the converter operation over a half switching cycle is completed.

## 4. Performance Comparison between NCDR and CCDR

This section will compare both the features and characteristics of NCDR as well as CCDR, which include secondary winding peak current of transformer, voltage gain, and output current ripple.

### 4.1. Performance of NCDR

From Figures 3 and 7, during one complete switching cycle, the secondary winding peak current *i*_sec(peak)_ can be expressed as follows:

(1) $$\begin{array}{c} i_{\text{sec}(\text{peak})}=\frac{I_{o}}{2}+\frac{\left(V_{\text{sec}}-V_{o}\right)}{8Lf_{s}}, \end{array}$$

where *L* = *L*_1_ = *L*_2_ = *L*_3_ = *L*_4_, *V*_sec_ is secondary voltage of transformer, and the voltage stresses of free-wheeling diodes can be expressed as follows:

(2) $$\begin{array}{c} V_{D_{r1}}=V_{D_{r2}}=\frac{V_{\text{sec}}+V_{o}}{2},\\ V_{D_{r3}}=V_{D_{r4}}=\frac{V_{\text{sec}}-V_{o}}{2}, \end{array}$$

where *V*_*o*_ is output voltage. By applying the volt-second balance principle to the auxiliary inductors and output filter inductors, the voltage gain of the proposed rectifier then can be derived as follows:

(3) $$\begin{array}{c} \frac{V_{o}}{V_{\text{sec}}}=\frac{D}{2}, \end{array}$$

where *D* is duty ratio of power switches. From Figure 7 again, by using the interleaved current of the output inductors *L*_3_ and *L*_4_, the output current ripple can be expressed:

(4) $$\begin{array}{c} i_{o(\text{ripple})}=\frac{\left(1-2D\right)V_{o}}{2Lf_{s}}, \end{array}$$

where *f*_*s*_ is switching frequency of power switches.

### 4.2. Performance of CCDR

From Figures 9 and 10, during one complete switching cycle, the secondary winding peak current *i*_sec(peak)_ can be expressed as follows:

(5) $$\begin{array}{c} i_{\text{sec}(\text{peak})}=\left(\frac{V_{\text{sec}}-nV_{o}}{n^{2}L}\right)DT_{S}\left(\frac{n_{2}}{n_{1}}\right)-\frac{V_{o}}{L}, \end{array}$$

where *L* = *L*_1_ = *L*_2_, and the voltage stresses of free-wheeling diodes can be expressed as follows:

(6) $$\begin{array}{c} V_{D_{r1}}=V_{D_{r2}}=\frac{V_{\text{sec}}}{n}, \end{array}$$

where *n* = (*n*_1_ + *n*_2_)/*n*_1_ is turns ratio of coupled inductors. By applying the volt-second balance principle to the auxiliary inductors and output filter inductors, the voltage gain of the proposed rectifier then can be derived as follows:

(7) $$\begin{array}{c} \frac{V_{o}}{V_{\text{sec}}}=\frac{2D}{n\left(1+n\right)}. \end{array}$$

From Figure 10 again, by combining the currents of *i*_*L*_1__ and *i*_*L*_2__, output current ripple can be determined as

(8) $$\begin{array}{c} i_{o(\text{ripple})}=\left[\frac{\left(1+n\right)}{2}-2D\right]\frac{V_{o}}{Lf_{s}}. \end{array}$$

**Figure 12:**
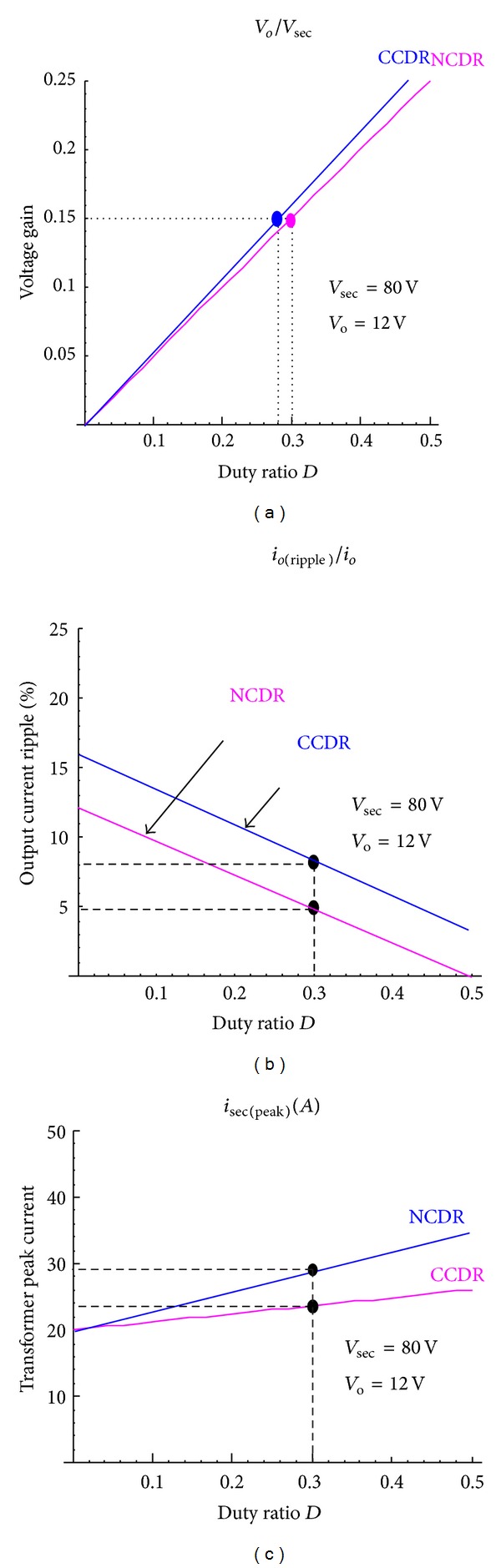
Performance comparison between NCDR and CCDR: (a) duty ratio, (b) output current ripple, and (c) secondary peak current of the transformer.

To objectively judge the merits and demerits of NCDR and CCDR, their performances are compared as summarized in Table 1 and Figure 12, assuming that the two improved current-doubler rectifiers can be operated with identical frequency, the same input and output voltages, and load currents.

## 5. Experimental Results

**Figure 13:**
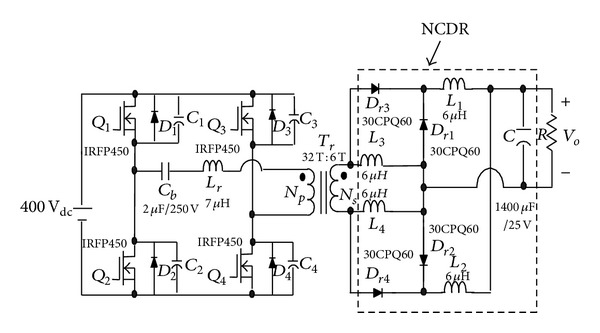
Experimental circuit of the phase-shift full-bridge converter with NCDR.

**Figure 14:**
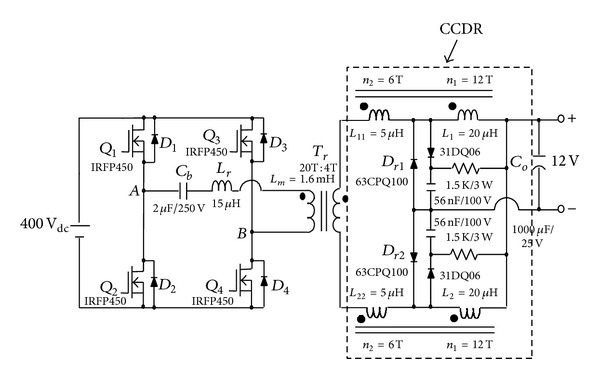
Experimental circuit of the proposed phase-shift full-bridge converter with CCDR.

To verity the performance of NCDR and CCDR, two sets of 500 W prototypes with phase-shift full-bridge converters were built (see Figures 13 and 14). The specifications are listed as follows:

- input voltage *V*_in_: 400 V_dc_,
- output current *I*_*o*_: 42 A,
- output voltage *V*_*o*_: 12 V_dc_.
- output power *P*_*o*_: 500 W,
- switching frequency *f*_*s*_: 100 kHz.

**Figure 15:**
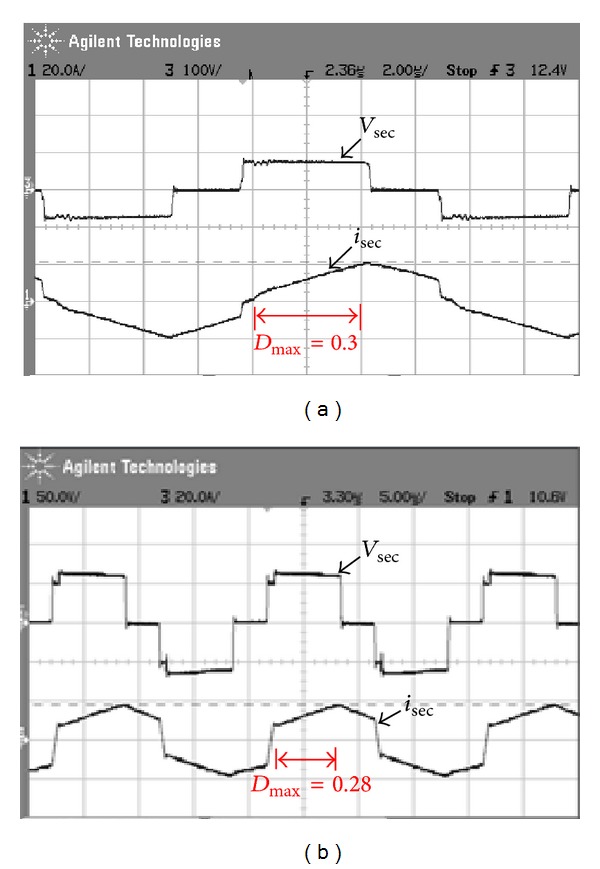
Measured waveforms of the secondary voltage and current of the transformer: (a) NCDR and (b) CCDR.

**Figure 16:**
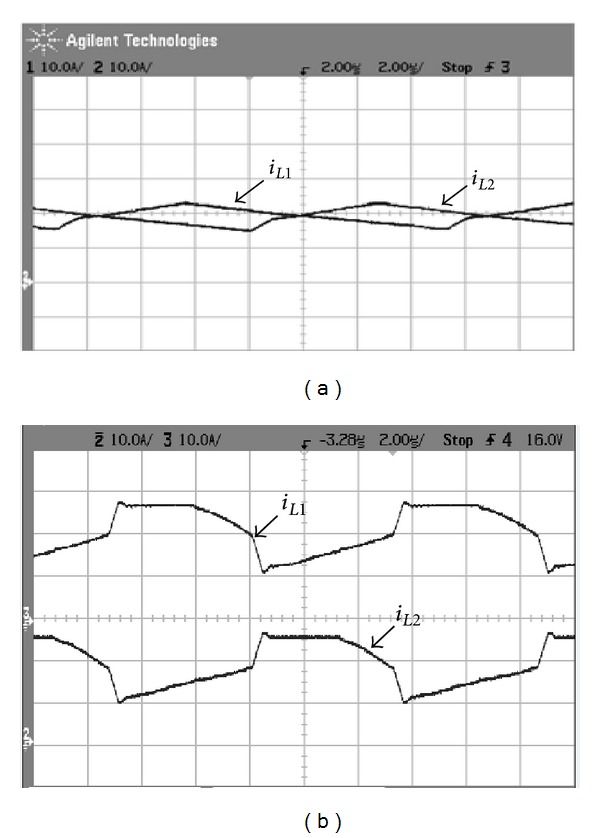
Measured waveforms of output filter inductor current *i*_*L*_1__ and *i*_*L*_2__: (a) NCDR and (b) CCDR.

**Figure 17:**
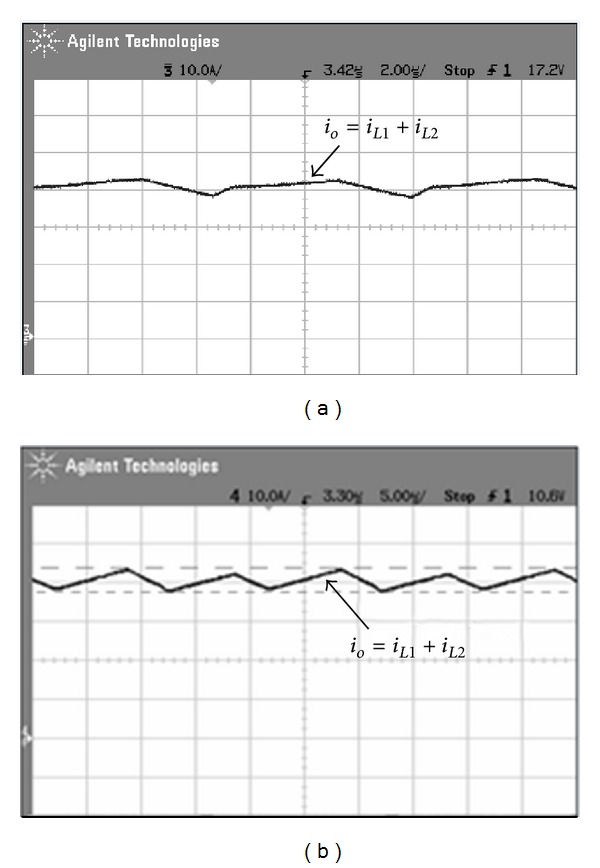
Measured output current *i*_*o*_ waveforms: (a) NCDR and (b) CCDR.

**Figure 18:**
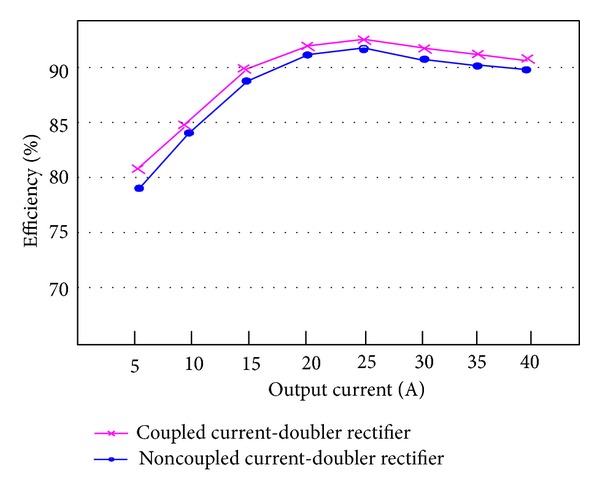
Efficiency comparison between NCDR and CCDR associated with phase-shift full-bridge converters.

Figure 15 shows measured transformer waveforms of NCDR and CCDR under full load condition. From these measured waveforms, it can be seen that NCDR and CCDR can be extended duty ratio. Comparing between NCDR and CCDR, the NCDR has a wide duty ratio.  Figure 16 shows waveforms of output filter inductors *L*_3_ and *L*_4_ for NCDR and CCDR, from which it can be seen that NCDR has lower inductor current ripple.  Figure 17 shows waveforms of full-load output current, from which it can be seen that NCDR has lower output current ripple.  Figure 18 shows the comparison of efficiency measurements between NCDR and CCDR, from which it can be seen that CCDR can achieve higher efficiency at heavy load and can reach as high as 91%. The reason behind is that NCDR is used with four inductors resulting in low conversion efficiency.

## 6. Conclusions

In this paper, the proposed phase-shift full-bridge converter with NCDR and CCDR under 500 W has been implemented. The NCDR has the merits of extended duty ratio, lower output current ripple, and lower rectifier diodes voltage stresses, which can reduce the peak current through the isolation transformer and switches. However, in comparison between efficiency of NCDR and CCDR, the NCDR has lower efficiency at full load condition. The reason behind is that NCDR is used with four inductors resulting in low conversion efficiency. For small size and high efficiency requirements, CCDR is relatively suitable for high step-down voltage and high power conversion applications.

## Figures and Tables

**Table 1:** Comparison between NCDR and CCDR.

	NCDR	CCDR
Voltage gain	$\frac{V_{o}}{V_{\text{sec}}}=\frac{D}{2}$	$\frac{V_{o}}{V_{\text{sec}}}=\frac{2D}{n(1+n)}$

Output current ripple	$i_{o(\text{ripple})}=\frac{(1-2D)V_{o}}{2Lf_{s}}$	$i_{o(\text{ripple})}=\left[\frac{\left(1+n\right)}{2}-2D\right]\frac{V_{o}}{Lf_{s}}$

Diode voltage stress	V_D_r1__ = V_D_r2__ = $\frac{V_{\text{sec}}+V_{o}}{2}$	V_*D*_*r*1__ = V_*D*_*r*2__ = $\frac{V_{\text{sec}}}{n}$

Peak current of transformer secondary winding	$i_{\text{sec}(\text{peak})}=\frac{I_{o}}{2}+\left(\frac{V_{\text{sec}}-V_{o}}{2L}\right)DT_{s}$	$i_{\text{sec}(\text{peak})}=\left(\frac{V_{\text{sec}}-{nV}_{o}}{n^{2}L}\right)DT_{S}\left(\frac{n_{2}}{n_{1}}\right)-\frac{V_{o}}{L}$

## References

[1] Zhang NF, Xu J (2011). A novel PCCM boost PFC converter with fast dynamic response. *IEEE Transactions on Industrial Electronics*.

[2] Huber L, Jang Y, Jovanović MM (2008). Performance evaluation of bridgeless PFC boost rectifiers. *IEEE Transactions on Power Electronics*.

[3] Cho J-G, Sabaté JA, Hua G, Lee FC (1996). Zero-voltage and zero-current-switching full bridge PWM converter for high-power applications. *IEEE Transactions on Power Electronics*.

[4] Xu M, Zhou J, Lee FC (2004). A current-tripler dc/dc converter. *IEEE Transactions on Power Electronics*.

[5] Jang Y, Jovanović MM, Chang Y-M (2003). A new ZVS-PWM full-bridge converter. *IEEE Transactions on Power Electronics*.

[6] Ruan X, Yan Y An Improved phase-shifted zero-voltage and zero-current switching PWM converter applied.

[7] Abu-Qahouq JA, Mao H, Batarseh I New coupled-inductors current-doubler topology.

[8] Chang LY, Chao KH, Chang TC (2012). Application of high voltage ratio and low ripple interleaved DC-DC converter for a fuel cell. *The Scientific World Journal*.

[9] Tsai C-T, Shen C-L (2013). High efficiency current-doubler rectifier with low output current ripple and high step-down voltage ratio. *IEEJ Transactions on Electrical and Electronic Engineering*.

[10] Tsai C-T, Shen C-L (2012). High step-down interleaved buck converter with active-clamp circuits for wind turbines. *Energies*.

[11] Tseng S-Y, Tsai C-T (2012). Photovoltaic power system with an interleaving boost converter for battery charger applications. *International Journal of Photoenergy*.

[12] Tsai C-T, Chen S-H (2012). PV power-generation system with a phase-shift PWM technique for high step-up voltage applications. *International Journal of Photoenergy*.

[13] Wu T-F, Tsai C-T, Chang Y-D, Chen Y-M (2008). Analysis and implementation of an improved current-doubler rectifier with coupled inductors. *IEEE Transactions on Power Electronics*.

